# Characterising the Tasmanian devil (*Sarcophilus harrisii*) pouch microbiome in lactating and non-lactating females

**DOI:** 10.1038/s41598-024-66097-8

**Published:** 2024-07-02

**Authors:** Lucy E. Ockert, Elspeth A. McLennan, Samantha Fox, Katherine Belov, Carolyn J. Hogg

**Affiliations:** 1 School of Life and Environmental Sciences, The University of Sydney, Sydney, NSW 2006 Australia; 2 Save the Tasmanian Devil Program, NRE Tasmania, Hobart, TAS 7001 Australia; 3 Toledo Zoo, 2605 Broadway, Toledo, OH 43609 USA; 4 Australian Research Council Centre of Excellence for Innovations in Peptide and Protein Science, The University of Sydney, Sydney, NSW 2006 Australia; 5 San Diego Zoo Wildlife Alliance, PO BOX 120551, San Diego, CA 92112 USA

**Keywords:** Marsupial, Skin, Reproduction, Immunology, Microbiome, Microbiome, Biodiversity, Genetics, Microbiology, Environmental sciences

## Abstract

Wildlife harbour a diverse range of microorganisms that affect their health and development. Marsupials are born immunologically naïve and physiologically underdeveloped, with primary development occurring inside a pouch. Secretion of immunological compounds and antimicrobial peptides in the epithelial lining of the female’s pouch, pouch young skin, and through the milk, are thought to boost the neonate’s immune system and potentially alter the pouch skin microbiome. Here, using 16S rRNA amplicon sequencing, we characterised the Tasmanian devil pouch skin microbiome from 25 lactating and 30 non-lactating wild females to describe and compare across these reproductive stages. We found that the lactating pouch skin microbiome had significantly lower amplicon sequence variant richness and diversity than non-lactating pouches, however there was no overall dissimilarity in community structure between lactating and non-lactating pouches. The top five phyla were found to be consistent between both reproductive stages, with over 85% of the microbiome being comprised of Firmicutes, Proteobacteria, Fusobacteriota, Actinobacteriota, and Bacteroidota. The most abundant taxa remained consistent across all taxonomic ranks between lactating and non-lactating pouch types. This suggests that any potential immunological compounds or antimicrobial peptide secretions did not significantly influence the main community members. Of the more than 16,000 total identified amplicon sequence variants, 25 were recognised as differentially abundant between lactating and non-lactating pouches. It is proposed that the secretion of antimicrobial peptides in the pouch act to modulate these microbial communities. This study identifies candidate bacterial clades on which to test the activity of Tasmanian devil antimicrobial peptides and their role in pouch young protection, which in turn may lead to future therapeutic development for human diseases.

## Introduction

The microbiome describes the community of microorganisms such as bacteria, fungi, and archaea that inhabit a particular environment, encompassing the interactions and genetic material of these microorganisms. The microbiome of a host and its relation to health and disease has been increasingly investigated in model organisms, particularly since the development and implementation of next-generation sequencing-based techniques^1^. Years of research into the human microbiome, initially influenced by the Human Microbiome Project, have revealed cautious links between disease and disruption of a healthy microbiome^2^. More recently, the microbiome has been investigated in non-model organisms such as livestock^3^ and aquaculture species^4,5^ due to their agricultural importance. The microbiome of wildlife species have also been investigated to understand novel challenges such as the impacts of captivity^6^, disease^7,8^, and to improve captive reproductive efforts^9,10^, as well as to explore the co-evolution of non-human hosts^11,12^ and the impact of diet and nutrition on the gut microbiome^13,14^. With global declines in biodiversity and an increased prevalence of human-wildlife interactions, recommendations have been made throughout the past decade to include microbial data in the recovery of threatened species^15^. The investigation of microbial communities in threatened species has previously aided our understanding of the effects of captivity^16^, habitat loss^17^, temperature changes^18^, exposure to chemicals in the environment^17^, and disease^8,19^ on the diversity of gut and skin microbiomes in wild species. This information has more recently been integrated into conservation strategies, with actions such as supplementing captive diets to increase microbial diversity in the gut^17,18^, or utilising the presence of anti-fungal bacteria on a host organism to treat disease in related species^20^. Understanding the microbiome in disease-free, wild animals has the potential to assist in therapeutic intervention when the normal diverse state of the microbiome is altered due to disease or captivity.

Marsupials give birth to physiologically underdeveloped young that attach to a teat for an extended period of time after a significantly shorter gestation period (ranging from 2 to 5 weeks) than their eutherian counterparts^21,22^. Marsupials are therefore exposed to a range of microorganisms at a much earlier stage of development than eutherian mammals. The marsupial pouch is an external abdominal fold of skin with a sphincter muscle enclosing the female’s teats and mammary glands. In some marsupial species, the pouch is where the developing young will remain for up to 80% of the total lactation period^23^ and provides protection by enclosing the young in a stable environment, free from predators and significant fluctuations in temperature and humidity^24^. The non-sterile pouch has been demonstrated to contain diverse microbial communities and bacteria that may be pathogenic to the developing young^25–32^. The internal pouch skin microbiome has so far been studied in six marsupial species to understand the mechanisms in place to protect the immunologically naïve young in a non-sterile pouch environment. These include the tammar wallaby (*Notamacropus eugenii*^27^), koala (*Phascolarctos cinereus*^30^), brushtail possum (*Trichosurus vulpecula*^32^) and quokka (*Setonix brachyurus*^33,34^), with studies using culture-dependent methods by inoculating culture medium with microbes obtained from moistened pouch swabs or pouch washes. More recently, the bacteria within the pouch of the southern hairy-nosed wombat (*Lasiorhinus latifrons*^29^), Tasmanian devil (*Sarcophilus harrisii*; hereafter, ‘devil’^28^), and koala (*Phascolarctos cinereus*^31^) has been characterised using 16S rRNA amplicon sequencing, a robust and cost-effective culture-independent method to identify bacterial units^35,36^. The abdominal skin and internal pouch microbiomes of devils were previously found to contain similar relative abundances of Firmicutes (skin—41.3%, pouch—36.2%) and Proteobacteria (skin—32.8%, pouch—34.4%) as the two dominant phyla at both sites^26^. The remaining 20% of both microbiomes were predominantly comprised of Bacteroidetes, Actinobacteriota, and Fusobacteria. Similarly, the skin and subadult (i.e., reproductively inactive) pouch microbiomes in southern hairy-nosed wombats exhibited microbial diversity and amplicon sequence variant (ASV) richness that was not statistically significantly different from each other^29^. During lactation, significant differences in microbial communities within the southern hairy-nosed wombat pouch were evident between reproductive stages, with pouches from lactating females containing less bacterial diversity than pouches from females without pouch young^29^. Bacterial communities in devil pouches were found to be highly dissimilar between different individuals that were lactating and those that were not, despite similarities in the overall bacterial diversity between pouch types, however this comparison was only performed for six samples from one location^28^. These previous findings suggest there is some form of modulation of the pouch microbiome during lactation.

At birth, immune tissues such as the spleen, thymus glands, and gut-associated lymphatic tissue, are structurally immature in marsupials and lack sufficient loads of immune cells to mount any significant adaptive immune response^37–39^. For example, dysbiosis in the koala pouch and high abundance of the bacterial species *Klebsiella pneumoniae* and *Pluralibacter gergoviae* have recently been associated with the mortality of koala pouch young^31^. Marsupials are proposed to have evolved a range of defence mechanisms to protect immunologically naïve young from infections^25^. It is believed that antimicrobial peptides (AMPs) secreted in the epithelial lining of the pouch alter the microbial community profile of the pouch in the presence of young^27,28,32,34,40^. As our study is focusing exclusively on the bacterial communities in the pouch given its preliminary nature in this area, any subsequent mentions of the microbiome are solely in reference to bacterial communities.

The Tasmanian devil is the largest extant carnivorous marsupial, with wild populations restricted to the island state of Tasmania, Australia. The outbreak of devil facial tumour disease (DFTD) in 1996^41^ and devil facial tumour 2 (DFT2) in 2014^42^ have caused declines of up to 80% among infected populations. Recent studies have suggested AMP families to be important components of the devil immune system^28^. Specific AMPs are expressed in the pouch lining and milk of the mother as well as the skin of devil joeys, likely providing immunological protection to underdeveloped pouch young^28^. A pilot study (n = 6) conducted by Peel et al.^28^ identified bacterial diversity to remain consistent across reproductive stages, yet the abundance of bacterial phyla was altered during lactation. Additionally, an earlier study characterised the pouch microbiome of reproductively inactive devils in intensively managed, semi-managed, and free-range populations, and found the pouch microbiome to be consistently highly similar to the skin in terms of observed Operational Taxonomic Units (OTUs), phylotype richness, and taxonomic composition, despite the significant community dissimilarity between captive and wild devils^26^. Due to the challenges of repeated wildlife sampling, differences in the lactating microbiome between intensively and extensively managed marsupial populations remains largely unknown. Temporal changes are beginning to be explored, however, with a recent study examining temporal changes in the microbiome of the koala pouch throughout the differing stages of pouch young development^31^.

The nature of collecting microbial samples from wildlife requires careful planning to mimic the ‘gold-standard’ protocols of model organism microbial studies. Despite the well documented protocols for researching the human microbiome, there are no standardised methods guiding the collection of microbiome samples from wildlife^43^. While wildlife protocols can take inspiration from human-based protocols, repeated sampling from the same individual is extremely challenging and unpredictable, making it difficult to assess temporal changes in wildlife microbiomes. Additionally, low sampling rates in many wildlife species means that the sampling of demographically similar individuals is often not feasible. As a result, samples are often collected from multiple geographic locations where factors such as diet, population structure, environmental conditions, exposure to external microorganisms, or exposure to anthropogenic disturbances increase variation between individuals^44–48^. These differences, however, can be accounted for by including location as a covariate in analyses where possible.

Here we build on previous investigations by utilising a larger sample size collected from wild, free-ranging devils. We aimed to characterise and compare the taxa and community diversity between the pouch microbiome of lactating and non-lactating devils.

## Methods

### Sample collection

**Figure 1:**
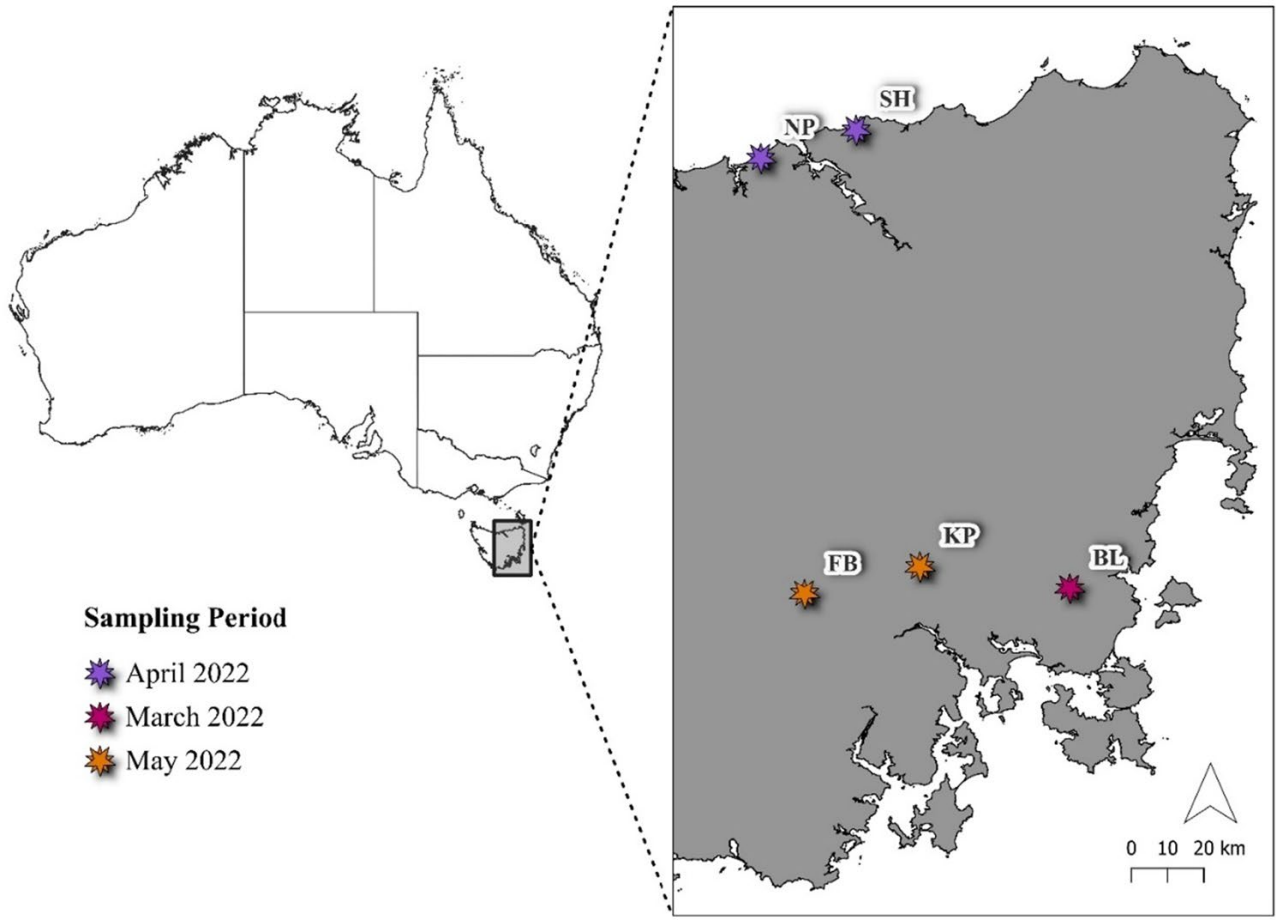
Map of sites where pouch swab samples were collected from Tasmanian devils. Samples were collected from the following sites for microbial analysis; Narawntapu National Park (NP; N = 11), Stony Head (SH; N = 5), Fentonbury (FB; N = 18), Kempton (KP; N = 16), and Buckland (BL; N = 4). A breakdown of the number of samples collected for each sampling group can be found in Additional file 1: Table S2. Colour corresponds to month when fieldtrip took place, scale bar represents kilometres, and arrow indicates north. Graphic was created using QGIS (v 3.22) and Australian Bureau of Statistics Digital Boundary Files^100^.

Sample collection trips were conducted by Save the Tasmanian Devil Program (STDP) and University of Sydney (USYD) staff during the 2022 breeding season (March–July^49^) across five locations (Fig. 1): Stony Head (41° 1′ 47.71 S, 146° 58′ 51.60″ E), Fentonbury (42° 37′ 27.06″ S, 146° 48′ 14.93″ E), Kempton (42° 31′ 44.94″ S, 147° 11′ 58.56″ E), Buckland (42° 36′ 16.79″ S, 147° 43′ 0.59″ E) and Narawntapu National Park (41° 7′ 34.80″ S, 146° 39′ 14.39″ E). Pouches were scored according to their reproductive appearance as described by Hesterman et al.^50^ (Additional file 1: Table S1). Pouch swab samples were collected from pro-oestrus (non-lactating; scores 3 and 4) females, and females with small (crown-rump length of < 50 mm) pouch young aged 70 days or less^51^ (lactating; pouch score 7). All females sampled did not have obvious outward signs of DFTD. A dry sterile swab (Promed^®^ swab aluminium stick with rayon tip) was wiped across the inner surface of the pouch three to five times, a suitable method for collecting microbial samples from the skin^28,29,31^.

Swabs were aseptically sealed in a sterile container and kept on ice in the field. They were then stored at – 20 °C prior to extraction. A total of 55 samples were used in this study; 25 from lactating females and 30 from non-lactating females. A power analysis was conducted in the *pwr* package^52^ in R (version 4.2.1^53^) to determine the minimum sample size to detect large differences (equivalent to an effect size of 0.8) in community composition (detected using UniFrac distance) between two equal sized sample groups; 25 samples per group gave a power of 0.8 to detect a change at a significance level of alpha = 0.05.

### DNA extraction and sequencing

Microbial DNA was extracted using the DNeasy PowerSoil Pro Kit (Qiagen, Hilden, Germany). Extractions were conducted according to the Qiagen protocol with the following modifications: (i) the rayon end of the swab was cut off into the PowerBead tube, and (ii) the microcentrifuge tube containing the lysis buffer (Qiagen solution CD1) and swab tip were homogenised for 10 min in an Omni Bead Ruptor 4 at 3 m/s to lyse the bacterial cells present on the swab. Negative control extractions in each extraction batch omitted the swab. Extraction products were tested for DNA quantity and purity using a NanoDrop 2000 Spectrophotometer (ThermoFisher Scientific, Waltham, MA, USA), and DNA concentration was quantified with a Qubit 2.0 Fluorometer using the Qubit dsDNA HS Assay Kit (ThermoFisher Scientific). An in-house PCR was performed on a small subset of samples to ensure that amplifiable microbial DNA was present (protocol outlined in Additional file 2). Extracted DNA was sent to the Ramaciotti Centre for Genomics (University of New South Wales, Kensington, Australia) for library preparation and sequencing. PCR products were indexed using the KAPA HiFi Hot Start Readymix (Roche, Basel, Switzerland), and normalised and pooled for sequencing using the SequalPrep™ Normalization Plate Kit (Invitrogen, Waltham, MA, USA) following the manufacturer’s instructions. 16 s rRNA V3-V4 amplicon sequencing was performed using the Illumina MiSeq v3 platform (2 × 300 bp) to amplify the V3-V4 hypervariable region in bacteria only. This region was selected for sequencing due to the higher rate of diversity capture as compared to primer pairs targeting other hypervariable regions^54^. Negative extraction controls were also sequenced. The standard V3-V4 primer pair, 341f (5ʹ–CCTACGGGNGGCWGCAG–3ʹ) and 805r (5ʹ–GACTACHVGGGTATCTAATCC–3ʹ)^55^, was used to generate sequencing libraries, with sequencing generating 300 bp paired-end reads.

### Data processing and statistical analysis

QIIME2 (version 2022.2^56^) was used to process and analyse the resulting raw reads. Demultiplexed paired-end sequence reads were merged, denoised, and quality filtered into ASVs using the DADA2 plugin^57^. Reads with a median quality score < Q30 were trimmed and truncated, producing trimmed reads that were 523 bp long. A feature table and phylogenetic tree were produced using the QIIME2 ‘feature-table summarize’ and ‘align-to-tree-mafft-fasttree’ commands, respectively. ASVs were assigned to taxonomy using a V3-V4 amplicon-specific naïve bayes classifier trained on the SILVA 138.1 small subunit (SSU) reference database^58^ using the QIIME2 plugin, RESCRIPt (Reference Sequence Annotation and Curation Pipeline^59^). An amplicon specific classifier was generated due to its improved accuracy in taxonomic classification of sequences^60^. The metadata table along with the resulting feature table, taxonomy table, and unrooted tree were imported and compiled into a phyloseq object using the *qiime2R* (version 0.99.6^61^) and *phyloseq* (version 1.40.0^62^) packages in R. Features present in the extraction controls were removed using the prevalence method in the *decontam* package (version 1.16.0^63^) at a threshold of 0.5 (Additional file 1: Figs. S3–S5). This threshold identifies ASVs as contaminants when their prevalence is higher in control samples than biological samples^63^. Additionally, mitochondrial, chloroplasts, other eukaryotic sequences, and ASVs classified under any kingdom other than bacteria were removed, as they are either not targeted by 16S sequencing and are therefore sequencing errors, or are likely to be artificially amplified while not actually contributing to the normal community composition^64^. Due to the unequal number of reads per sample, the feature table was normalised through rarefaction to a depth of 18,198 for diversity analysis.

Prior to conducting the focal analysis, exploratory analysis was undertaken to investigate any potentially confounding factors present in the dataset. A Welch’s t-test was used to assess any significant differences in the age of the female devils between reproductive groups. An exact date of birth cannot be measured for wild devils, so female age in years was used, knowing that sexual maturity is reached at approximately two years of age^49^. A similar data exploration could not be conducted for location due to the categorical nature of the data. Instead, location was included as a covariate in all steps of analysis.

The relative abundance of ASVs were assessed per sample at different taxonomic ranks to visualise differences in dominant taxa and their relative abundance between sample groups. Additionally, taxa were agglomerated separately at each taxonomic level using the ‘tax_glom’ *phyloseq* function to extract relative abundance data per sample group. Alpha diversity (i.e., the diversity contained within a single sample) was assessed to compare the diversity of individual samples within each sample group. Observed ASVs^65^, Shannon Diversity Index^66^, and Faith’s Phylogenetic Diversity^67^ were selected as metrics to measure alpha diversity. The statistical significance of these metrics was compared between reproductive statuses and locations using generalised linear models (GLMs), with the response variable, alpha diversity, tested against the predictor variables of reproductive status and location.

Beta diversity (i.e., the similarity or dissimilarity between two communities) was assessed using weighted and unweighted UniFrac distances and visualised by Non-Metric Multidimensional Scaling (NMDS). UniFrac distance was selected over other beta diversity metrics as it measures the dissimilarity between microbial communities based on the unique and shared evolutionary history of their constituent species^68^. Both UniFrac distance matrices were used to determine if compositional differences were present, and if so, whether they were influenced by the abundance of taxa. Statistical significance of UniFrac dissimilarity between sample groups was tested using a Permutational multivariate analysis of variance (PERMANOVA) test with 999 permutations through the *vegan* package (version 2.6-2^69^) in R. The homogeneity of group dispersions was tested using the ‘betadisper’ function from the *vegan* package as it may influence PERMANOVA results (Additional file 1: Fig. S1). Additionally, pairwise multilevel comparisons were conducted using the *pairwiseAdonis* package (version 0.4^70^) to determine the spatial drivers of community dissimilarity. Due to the unequal sample sizes at some locations, we also conducted a pairwise comparison between reproductive status and beta diversity within the sites with larger sample sizes (i.e. Fentonbury, Kempton, and Narawntapu National Park). This approach was chosen instead of an overall comparison between all locations due to non-homogenous dispersion of unweighted UniFrac between locations. The non-homogenous group dispersions may confound any outcomes of an overall comparison.

Differentially abundant taxa were identified and statistically compared using *DESeq2* (version 1.36.0^71^) in R. This differential abundance analysis was conducted on non-rarefied sequencing data due to the alternate normalisation methods utilised by *DESeq2*^72^. Testing was conducted to determine differentially abundant taxa between lactating and non-lactating pouch microbial communities at multiple taxonomic ranks; phylum, class, order, family, genus, and ASVs. ASVs were agglomerated into taxonomic groups prior to analysis using *phyloseq*’s ‘tax_glom’ function, hence ASVs were chosen instead of species to avoid agglomeration of unidentified species.

### Ethics declarations

All research involving the capture, handling, and collection of wild samples was carried out in accordance with guidelines under the University of Sydney Animal Care and Ethics Committee and all collection protocols were approved by the University of Sydney Animal Care and Ethics Committee approval number 2019/1562. As the subjects of the sample collection were wild animals no informed consent was required. Samples collected for this study were collected by the Tasmanian Department of Natural Resources and Environment’s Save the Tasmanian Devil Program under their scientific permit.

## Results

The average age of devils was significantly higher among lactating individuals (mean ± SD, 2.24 ± 0.78 years) as compared to non-lactating individuals (1.13 ± 0.35; t = 6.5841, p < 0.0001; Additional file 1: Fig. S2).

A total of 62 samples (55 devils + 7 negative extraction controls) were sequenced, resulting in 3,344,445 total reads. After filtering, trimming, decontamination, and removal of non-targeted taxa, 16,106 ASVs were identified throughout an average of 59,940 reads and a median of 56,189 reads per sample. The sequencing of the negative controls yielded a total of 1397 reads (an average of 200 reads per control) following which 12 ASVs were identified as contaminants and removed from the dataset before further analysis (Additional file 1: Table S4). Some of the contaminating genera identified by *decontam* are common contaminants of lab reagents and disposables, such as *Burkholderia*, *Pseudomonas*, and *Streptococcus*^73–75^.

**Figure 2:**
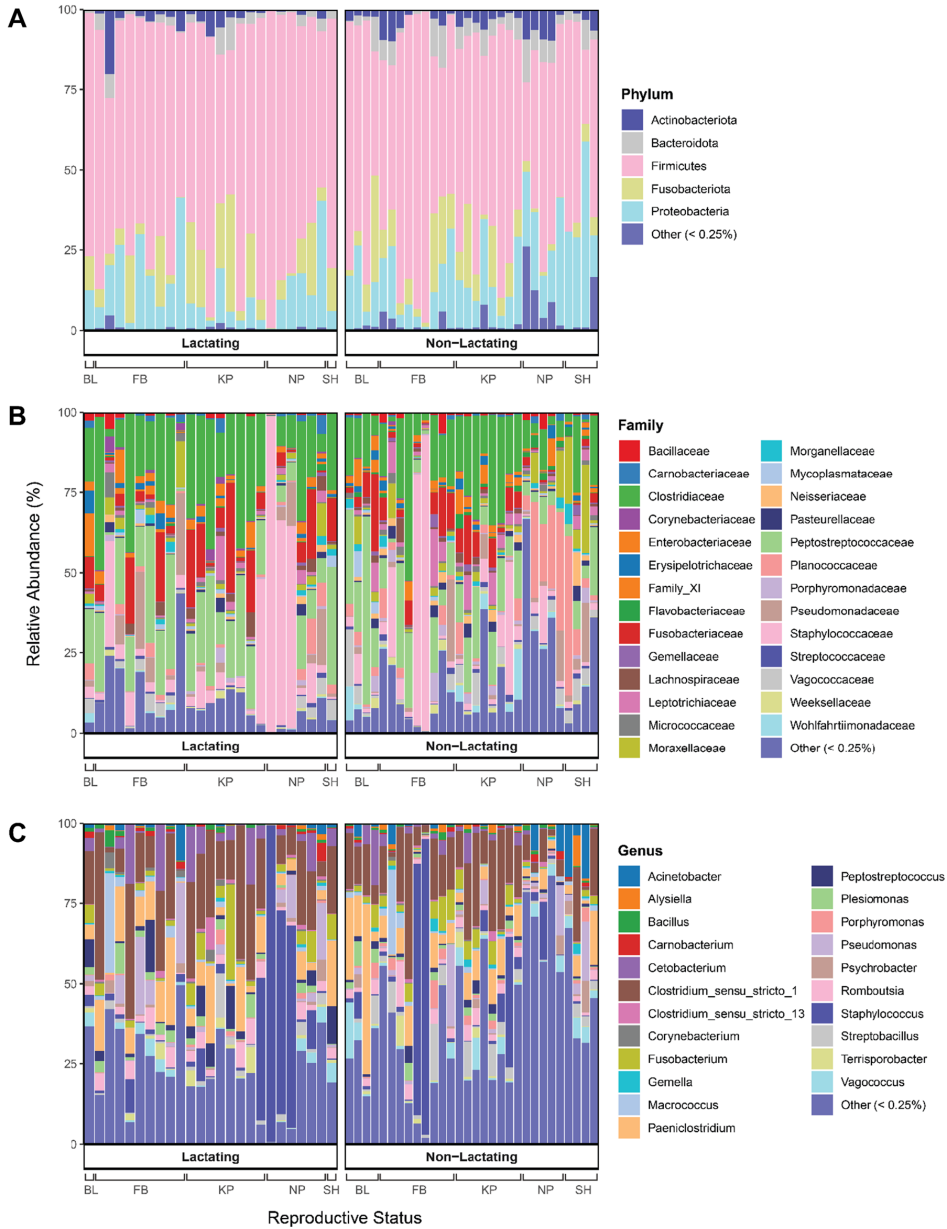
Taxonomy bar plots displaying the relative abundance of bacteria present in Tasmanian devil pouches across reproductive stages at three taxonomic levels; (**A**) Phylum, (**B**) Family, and (**C**) Genus. Each column represents one sample. Samples are organised according to reproductive stages; lactating and non-lactating. Location is indicated below the x-axis as follows; BL = Buckland, FB = Fentonbury, KP = Kempton, NP = Narawntapu National Park, and SH = Stony Head. Taxa with a median relative abundance of < 0.25% were merged into ‘Other’. Visualisation was performed using *ggplot2* (v 3.4.2).

The main taxa identified in the pouch microbiome were consistent across reproductive groups (Fig. 2). The relative abundance of taxa in each pouch type across all ranks is included as Additional file 4. Over 96% of the bacteria in devil pouches was composed of five phyla; Firmicutes (L—73%, NL—61%), Proteobacteria (L—12%, NL—17%), Fusobacteriota (L—10%, NL—9%), Actinobacteriota (L—3%, NL—4%), and Bacteroidota (L—2%, NL—5%) (Fig. 2A). Furthermore, 70% of the microbiota from lactating pouches consisted of ASVs from five families; Staphylococcaceae (22%), Clostridiaceae (20%), Peptostreptococcaceae (16%), Fusobacteriaceae (8%) and Pseudomonadaceae (4%). The same five dominant families, however, comprised only 46% of the non-lactating devil pouch microbiome; Staphylococcaceae (12%), Clostridiaceae (16%), Peptostreptococcaceae (13%), Fusobacteriaceae (5%) and Pseudomonadaceae (3%) (Fig. 2B). Additional families that had higher relative abundances in non-lactating pouches and may be identified as dominating in this pouch type are Planococcaceae (7%), Moraxellaceae (3%), Leptotrichiaeceae (3%), and Vagococcaceae (3%) (Additional File 4). Firmicutes species comprised the largest proportion of the pouch microbiome, with 38% (L) and 56% (NL) of the 10 most abundant genera classified as Firmicutes; *Staphylococcus* (L—20%, NL—10%), *Clostridium *sensu stricto* 1* (L—18%, NL—14%), *Paeniclostridium* (L—9%, NL—8%), *Brochothrix* (L—3%, NL—1%), *Romboutsia* (L—3%, NL—3%), *Peptostreptococcus* (L—3%, NL—1%), and *Macrococcus* (L—2%, NL—2%) (Fig. 2C). Other dominating genera included *Cetobacterium* (L—4.7%, NL—2.3%), *Pseudomonas* (L—4.2%, NL—2.7%), and *Fusobacterium* (L—4.7%, NL—2.3%).

### Sample diversity

**Figure 3:**
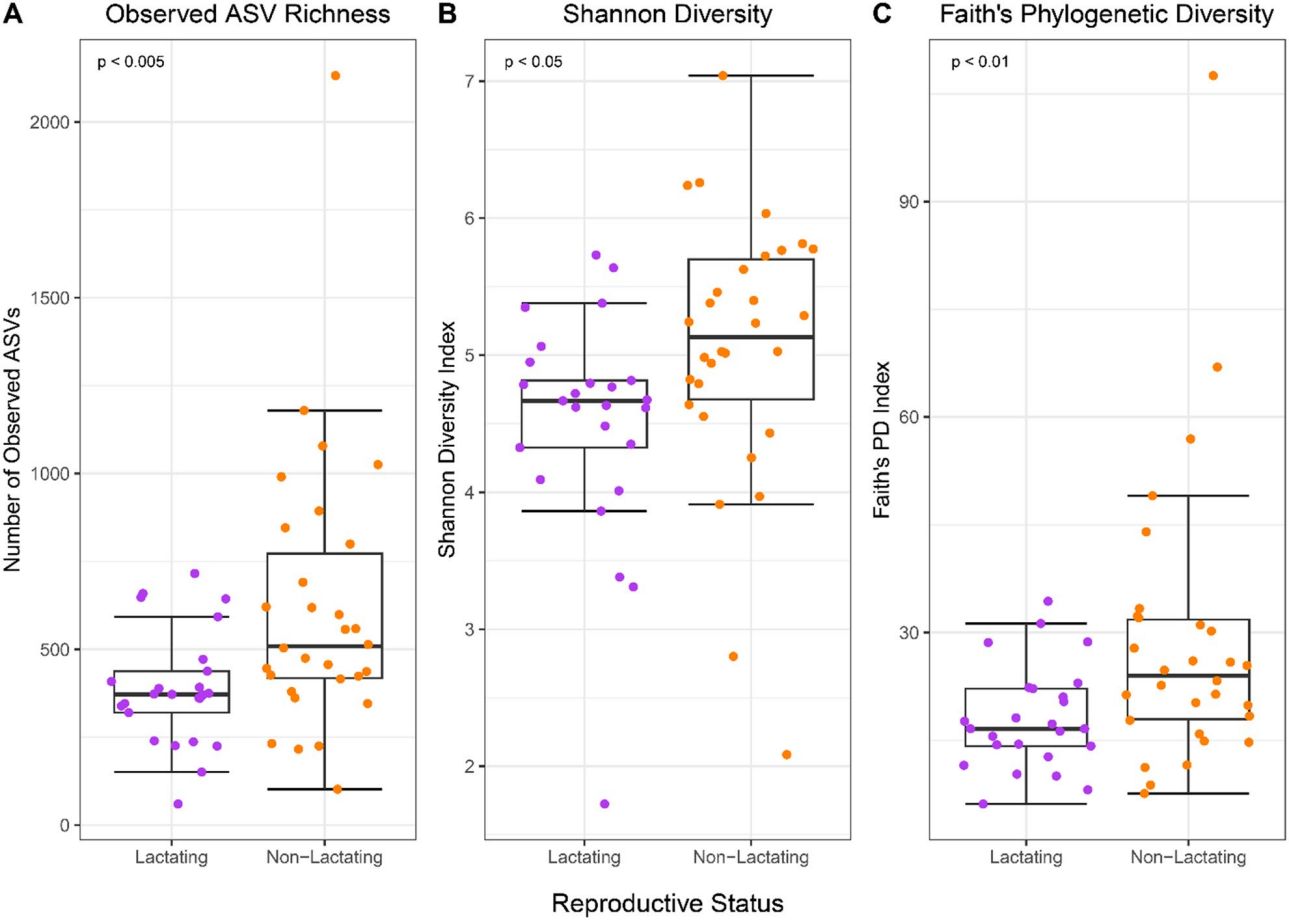
Alpha diversity metrics of pouch microbial compositions per reproductive status. Whisker box plots depict (**A**) Observed Amplicon Sequence Variant Richness, (**B**) Shannon Diversity, and (**C**) Faith’s Phylogenetic Diversity from lactating (purple; n = 25) and non-lactating (orange; n = 30) devils. Results of statistical analysis can be found in Additional file 1: Table S5. Visualisation was performed using *ggplot2* (v 3.4.2).

Alpha diversity measures revealed the pouches of lactating devils to have a significantly lower observed richness present (Observed ASVs; 389 ± 163) than in the pouches of non-lactating devils (618 ± 392; t = 2.99, *p* < 0.005; Fig. 3A; Additional file 1: Table S5A). Lactating pouches also exhibited lower community diversity (Shannon Diversity Index; 4.5 ± 0.8) than non-lactating pouches (5.1 ± 1.0; t = 2.11, *p* < 0.05; Fig. 3B; Additional file 1: Table S5B). The phylogenetic diversity was also lower in lactating pouches (Faith’s Phylogenetic Diversity; 18.1 ± 7.2) compared to non-lactating pouches (28.8 ± 20.2; t = 2.81, *p* < 0.01; Fig. 3C; Additional file 1: Table S5C). Samples from Narawntapu National Park, regardless of reproductive status, were identified as having significantly more ASVs (t = 2.06, *p* < 0.05) and significantly higher phylogenetic diversity (t = 2.31, *p* < 0.05; Additional file 1: Table S5).

### Community diversity

**Figure 4:**
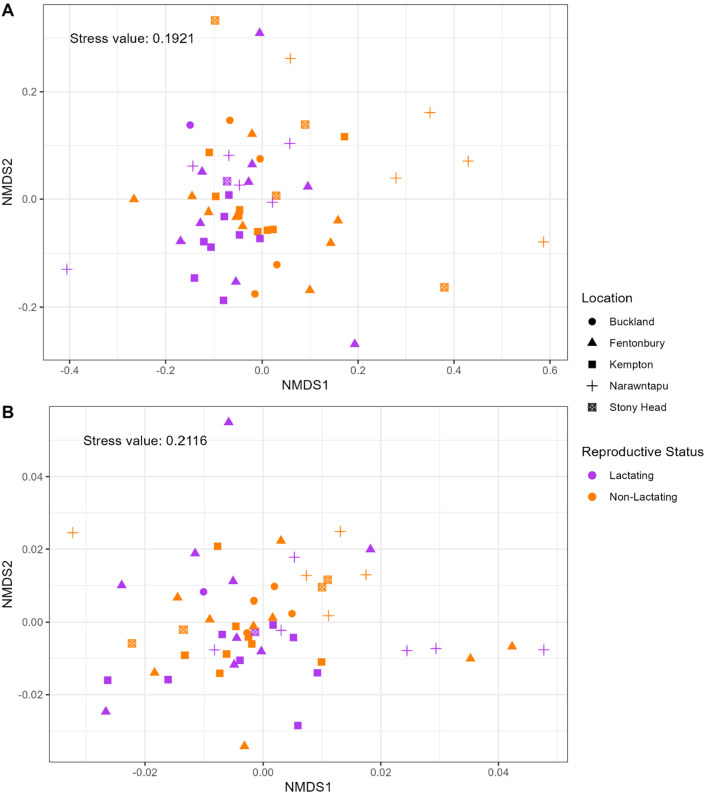
Differences in microbial community composition observed between reproductive status and location. Nonmetric Multidimensional Scaling (NMDS) plots of (**A**) unweighted, and (**B**) weighted UniFrac distances. Colour represents reproductive status while shape indicates location. Results of statistical analysis can be found in Additional file 1: Table S6. Visualisation was performed using *ggplot2* (v 3.4.2).

While no distinct clustering is visible, the unweighted UniFrac NMDS plot indicates non-lactating samples to be more spread out than lactating samples (Fig. 4A). A PERMANOVA was used to determine if this distinction between the two reproductive groups was statistically significant. Unweighted UniFrac distance was significantly different between reproductive statuses (F = 1.84, *p* < 0.05) and locations (F = 1.20, *p* < 0.005; Additional file 1: Table S6A), however, the significant difference in dispersion (reproductive status: F = 5.86, *p* < 0.05; location: F = 8.31, *p* < 0.005; Additional file 1: Table S7) means that it cannot be concluded whether the significant difference in unweighted UniFrac distance is due to average community structure or variation in dispersion between groups (Additional file 1: Table S7A; Table S7B). Weighted UniFrac distance, however, was significantly different between locations (F = 1.75, *p* < 0.005) but not reproductive status (F = 0.87, *p* > 0.05; Fig. 4B; Additional file 1: Table S6B). The dispersion effects for weighted UniFrac distance were not significantly different (reproductive status: F = 3.67, *p* = 0.065; location: F = 1.15, *p* = 0.346; Additional file 1: Table S7). Due to the high NMDS stress score and unclear unweighted UniFrac results, a PCoA was run yet also failed to identify any clear clustering between treatment groups (Additional file 1: Fig. S6). The pairwise comparisons of UniFrac distance between reproductive status within each location separately found there to be a significant difference in the community composition of the pouch microbiome between lactating and non-lactating females at Narawntapu National Park (unweighted UniFrac: F = 1.40, *p* < 0.01; weighted UniFrac: F = 2.547, *p* < 0.05; Additional file 1: Table S8, Fig. S7), but the microbial communities in the pouches of lactating and non-lactating females from Fentonbury and Kempton were not dissimilar from each other (Additional file 1: Table S8).

**Figure 5:**
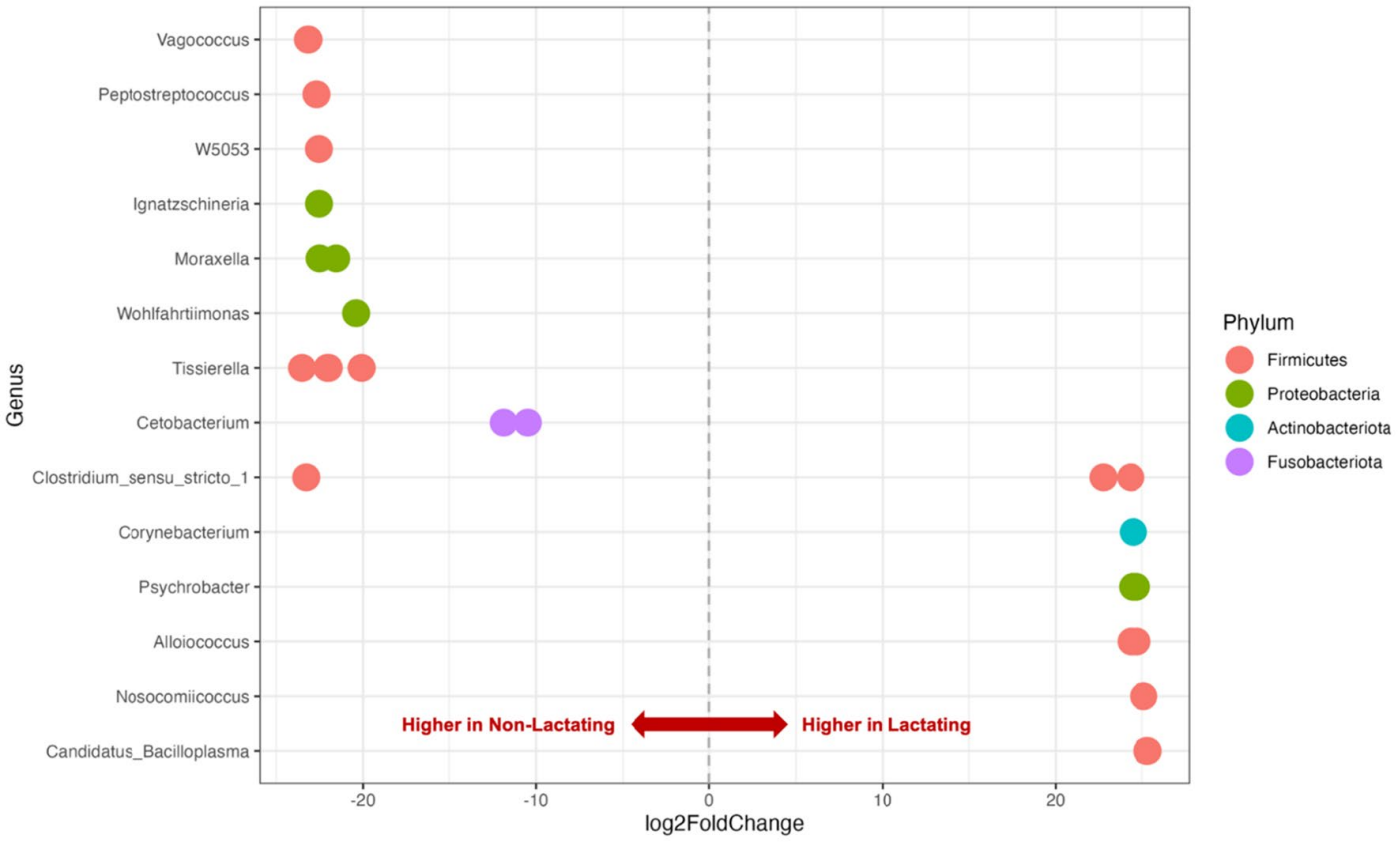
Differentially abundant amplicon sequence variants (ASVs) between reproductive groups as identified by DESeq2 analysis. Each point represents a single differentially abundant ASV, grouped according to genus. The comparison was made between lactating versus non-lactating pouches, hence a positive Log_2_ Fold Change indicates a higher prevalence in lactating pouches and a lower prevalence in non-lactating pouches. All ASVs visualised above were found to be significantly different between reproductive groups using a Kruskal–Wallis test. Visualisation was performed using *ggplot2* (v 3.4.2).

The results from the DESeq2 differential abundance analysis found four orders, three families, one genus, and 25 ASVs to be differentially abundant between lactating and non-lactating pouches (Fig. 5; Additional file 1: Table S9). Of the 25 ASVs identified as differentially abundant, 16 were from the phylum Firmicutes, six Proteobacteria, two Fusobacteriota, and one Actinobacteriota (Fig. 5). *Clostridium* and *Tisierella* ASVs were most commonly identified as differentially abundant. *Clostridium botulinum* and *Clostridium paraputrificum* were more abundant in lactating pouches, whereas *Clostridium colicanis* was more abundant in non-lactating pouches. Four unidentified *Tisierella* ASVs were more abundant in non-lactating pouches than lactating. Apart from *Clostridium* spp., ASVs from the same genus exhibited consistent positive or negative log2 fold changes. All ASVs identified as differentially abundant produced a BH adjusted p-value of < 0.0001 (Additional file 1: Table S9).

## Discussion

Here, we aimed to characterise the microbiome of pouches from lactating and non-lactating female Tasmanian devils and to identify differences in ASV richness and diversity between the two groups. We found lactating and non-lactating pouches to consist of the same dominating taxa, yet lactating pouches contained fewer overall ASVs and were less diverse. We demonstrate the dominating phyla to be Actinobacteriota, Bacteroidota, Firmicutes, Fusobacteria, and Proteobacteria, comprising a combined total of 99% and 96% of the microbiome in lactating pouches and non-lactating pouches, respectively. Previous studies found the same five phyla to comprise ~ 90% of the microbiome in reproductively inactive devils^26^, while the only previous study to investigate the pouch microbiome of lactating devils did not report relative abundances at the phylum-level^28^. Our study had different average relative abundance in non-lactating pouches to previous studies for Firmicutes, 61.3% here compared to 28.4%^28^ and 36.2%^26^, and Proteobacteria, 17.2% here compared to ~ 35% previously^26,28^. The relative abundance of Fusobacteria was within 1% of the relative abundance in non-lactating pouches as identified by Cheng et al. yet was ~ 18% lower than the relative abundance identified by Peel et al. The mean relative abundances of Bacteroidota and Actinobacteria in non-lactating pouches were within 3% of values from previous findings^26,28^. The differences observed in abundance of Firmicutes and Proteobacteria may be due to either differing analysis techniques between our study and previous work, or environmental conditions experienced by sampled individuals. Peel et al.^28^ and Cheng et al.^26^ clustered their sequences into OTUs, whilst we utilised ASVs, a more reproducible and contemporary method of denoising Illumina amplicon data^65^. OTUs and ASVs have been found to be comparable at higher taxonomic ranks, however, and exhibit highly similar alpha and beta diversity metrics, producing similar ecological conclusions^76–78^. As Peel et al.^28^ utilised V3-V4 amplicons sequenced on an Illumina MiSeq, as per this study, differences in analysis are unlikely to be driving the differences observed with the relative abundance of Firmicutes and Proteobacteria. The most likely explanation of the differences observed between our study and previous ones, is environmental variation between sampled individuals. Differences in results may also be influenced by the stage of lactation at which samples were collected. It is well documented that the immunological properties of marsupial milk can change throughout the stages of pouch young development (see^79,80^ and references therein), and as such, the expression of antimicrobial peptides in the pouch may also fluctuate throughout lactation, yet the stage at which samples were collected for Peel et al.^28^ is unknown. Here we sampled a greater number of individuals across multiple geographic locations, likely to be more indicative of the true patterns of microbial communities within the devil pouch. Cheng et al.^26^ found microbial communities present in the pouch of captive devils to be significantly different to wild devils, with significant variation between some wild sites (*N* = 18). Just as Cheng et al.^26^ identified differences in microbial communities of devil pouches across geographic locations, we identified samples from Narawntapu National Park to exhibit higher ASV richness and phylogenetic diversity. Narawntapu National Park was also the only location where the overall microbial communities associated with lactating and non-lactating pouches were significantly dissimilar from each other. To truly disentangle spatial differences in pouch microbiome, future studies should sample between geographic locations at the same time point to remove any seasonal breeding effects. Furthermore, it is uncertain whether the lower number of observed ASVs and diversity found in the lactating pouch were driven by lactation or by the age of the female devil. Since sampling took place during the breeding season at in-situ sites with small populations of devils, most of the adult females were reproductively active, making it challenging to collect samples from females of the same age to eliminate this variation. This is a limitation of this study, and future studies may be able to address this by collecting samples for lactating females during the breeding season and for non-lactating females outside of the breeding season.

The previous study investigating changes in the pouch microbiome associated with lactation in wild devils found the level of diversity in pouches from both reproductive stages to be ‘similar’, yet differences in abundance caused lactating pouches to exhibit a high degree of dissimilarity from non-lactating pouches^28^. This differs from our results which found a significant difference in species diversity between the microbiome of lactating and non-lactating pouches but failed to identify distinct community differences. Similarly, recent studies comparing the pouch microbiome of lactating and non-lactating pouches in koalas and southern hairy nosed wombats found significant differences in species diversity and richness, however, unlike these studies, we failed to identify distinct community differences between lactating and non-lactating pouches^29,31^. The lower species richness and diversity observed here between lactating pouches and non-lactating pouches indicates a reduction in the number and type of species present. The lower phylogenetic diversity means that the bacterial species present in the presence of pouch young are more closely related. This reduction in richness and diversity may be due to the proposed antimicrobial action of pouch secretions^28,81–83^. Peel et al.^28^ identified one antimicrobial peptide (cathelicidin, Saha-CATH2) as being expressed more highly in the pouch versus other tissues. This cathelicidin, however, showed minimal antimicrobial activity against the bacterial strains tested, some of which were found in the devil pouch, such as *Staphylococcus aureus* and *Pasteurella multocida.* Instead, it is possible that Saha-CATH2 may be active against bacteria that was not tested but is found at higher relative abundances in the non-lactating devil pouch (i.e. *Clostridium* spp.). In addition, Saha-CATH2 was found to be evolutionarily related to tammar wallaby and opossum cathelicidins^28^. A closely related tammar wallaby cathelicidin, MaeuCath8, was highly expressed in the female’s pouch, suggesting that secretions from epithelial cells within the pouch may provide antimicrobial protection to pouch young^84^. The decreased phylogenetic diversity of microbial pouch communities during lactation may be explained by the expression of antimicrobial peptides exhibiting specific antibacterial action against specific bacterial taxa within the pouch. Future studies testing the antimicrobial properties of devil pouch secretions should be guided by the bacterial genera that were found to be significantly lower in lactating devil pouches; *Vagococcus*, *Peptostreptococcus*, *W5053*, *Ignatzschineria*, *Moraxella*, *Wohlfahrtiimonas*, *Tissierella*, *Cetobacterium*, and *Clostridium sensu strico 1.*

Many of the dominating phyla identified in the pouch microbiomes are highly prevalent in the skin microbiome of other vertebrates, with Actinobacteria, Firmicutes, Proteobacteria and Bacteroides dominating 99% of the human skin microbiome^85^. When comparing the devil pouch microbiome to the skin microbiome of other terrestrial mammals, it is evident that Firmicutes comprised a much larger proportion of the devil microbiome (60–70%) as compared to other terrestrial mammalian species such as the wild bank vole (*Myodes glareolus;* 28% Firmicutes^47^) or microbat species, which typically saw Actinobacteria as the most abundant phyla^86^. The relative abundance of Firmicutes is high in the Tasmanian devil pouch, even compared to other marsupial species such as the southern hairy nosed wombat (> 10–35% in reproductively active and inactive pouches, respectively) and the koala (10–12% during anoestrus and early lactation).

Multiple genera identified within the pouch, irrespective of reproductive status, are implicated with human and wildlife diseases, posing a potential threat to the immunologically naïve pouch young. *Vagococcus* contains two species that are pathogenic to domestic mammals, birds, and fish^87,88^, while *Peptostreptococcus* spp. have been identified in human skin diseases^89^. *Ignatzschineria* and *Wohlfahrtiimonas* spp. are pathogenic species that are interestingly both associated with the microbiome of maggot-infested wounds in humans^90,91^. Other differentially abundant taxa have been identified in the mammalian gastrointestinal tract, such as *Tissierella* and *Clostridium colicanis*^92–94^. *Tissierella* has been associated with some infections in humans^92^, while the pathogenicity of *C. colicanis* has not been well-explored. Additionally, increased abundance of *W5053* spp. in the mammalian urogenital tract has been associated with adverse reproductive conditions^95,96^. The decreased abundance of this genus in the pouches of lactating devils may illustrate a healthy reproductive environment. While some protection appears to be provided to the pouch young through the hypothesised mechanisms of antimicrobial secretions, some species of bacteria found in the lactating pouch are still potentially pathogenic. Species such as *Clostridium botulinum* and *Corynebacterium urealyticum* were more abundant in lactating pouches but are associated with paralysis and urological diseases in other vertebrates, respectively^97,98^. The impact of these species on the health of the immunologically naïve pouch young is unknown, yet with a 75% survival rate of pouch young to weaning^99^, it is likely that these bacteria are either non-pathogenic to this species or that other mechanisms of protection are employed by the female to protect the pouch young, potentially via antimicrobial peptides and/or the transfer of immunological compounds in the milk^79^.

## Conclusions

This study is one of the largest investigations into the microbial changes associated with lactation in the marsupial pouch. Our findings show that the bacterial communities found in lactating devil pouches are comprised of taxa that are more closely related and contain less rare taxa when compared to pouches of non-lactating devils. We propose that these results may in part be due to the secretion of antimicrobial peptides that are secreted by the pouch skin. Further investigation into the antimicrobial action expressed in the devil pouch against the suite of bacteria characterised here will determine the bacterial taxa targeted by these protective mechanisms, furthering our understanding of the immunological development of marsupial pouch young.

### Supplementary Information

Supplementary Information 1.

Supplementary Information 2.

Supplementary Information 3.

Supplementary Information 4.

Supplementary Information 5.

## Data Availability

The demultiplexed data generated and analysed during this study has been uploaded to the NCBI Short Read Archive (SRA) repository under BioProject PRJNA1063750. Code developed for analysis in QIIME2 and R Studio can be found at https://github.com/l-ockert/devil-pouch-microbiome.
