# Supplementary material for: Characterising the Tasmanian devil (Sarcophilus harrisii) pouch microbiome in lactating and non-lactating females
Source: Sci Rep. 2024 Jul 2;14:15188. doi: 10.1038/s41598-024-66097-8 (PMC11220038; doi:10.1038/s41598-024-66097-8)
Supplement: Supplementary file 1 — Supplementary Information 1. [file 41598_2024_66097_MOESM1_ESM.docx]

**Table S1.** Description of Tasmanian devil pouch appearance and reproductive status. The full spectrum of pouch scoring ranges from 1 (immature) – 9 (regressing), yet only three scores were selected as targets for this study. Pouch scores 3 and 4 were grouped for analysis as both statuses indicate pouches that are reproductively active but non-lactating, while score 7 indicates lactation and the presence of newly born pouch young. Pouch scoring methods and characteristics are derived from Hesterman et al. [47].

| Pouch Score | Pouch Appearance | Reproductive Status |
| --- | --- | --- |
| 3 | Droplets of greasy red exudate present. | Pro-oestrus (non-lactating) |
| 4 | Puffy with ‘lipstick ring’ around pouch border, greasy red exudate present. | Oestrus (non-lactating) |
| 7 | Studded tissue with small, hairless pouch young attached to teat | Early lactation (lactating) |

**Table S2.** Summary of number of samples per reproductive status at each site.

|  | Lactating | Non-Lactating |
| --- | --- | --- |
| Buckland | 1 | 4 |
| Fentonbury | 9 | 9 |
| Kempton | 8 | 8 |
| Narawntapu National Park | 6 | 5 |
| Stony Head | 1 | 4 |
| Total | 25 | 30 |


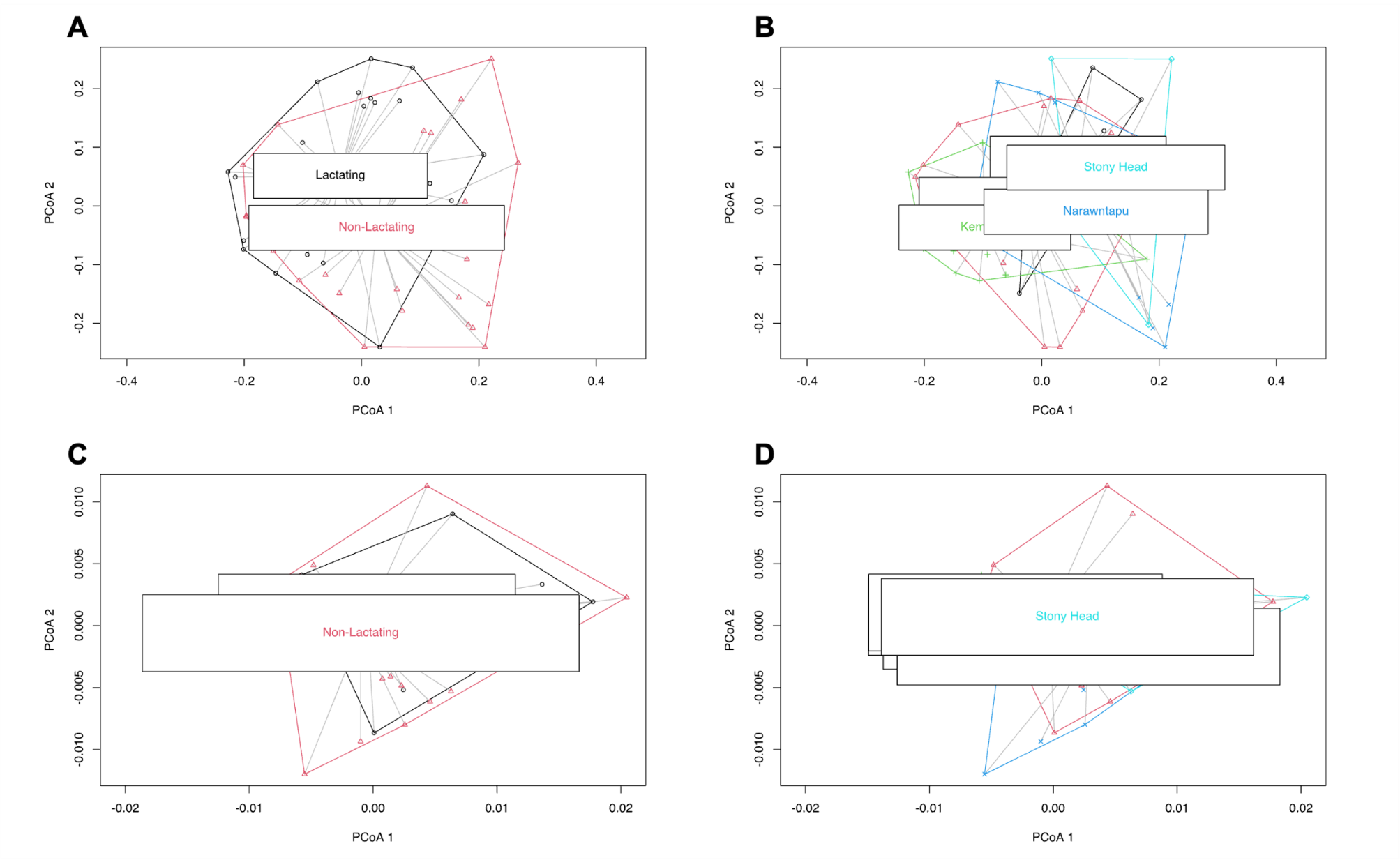
**Figure S1.** Centroid plots of UniFrac distances to visualise homogeneity of group dispersions. Plots are based on (A) Unweighted UniFrac distance between reproductive statuses, (B) Unweighted UniFrac distances between locations, (C) Weighted UniFrac distances between reproductive statuses, and (D) Weighted UniFrac distances between locations.


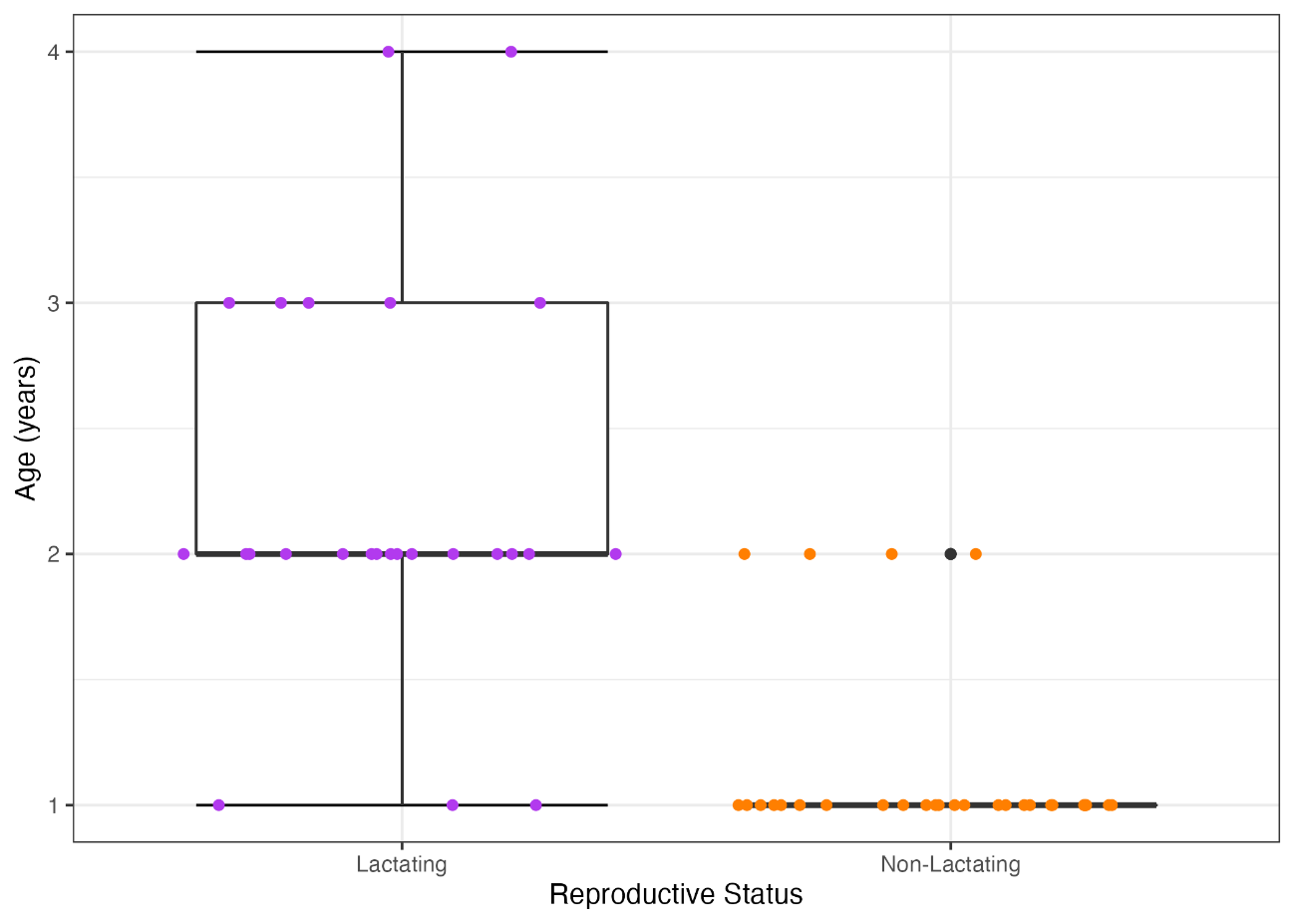


**Figure S2.** Distribution of the age of female Tasmanian devils sampled for pouch microbiome per reproductive stage. Single points represent individual samples while box and whisker plots display the distribution of age within each sampling group. A breakdown of the average age from each location per reproductive group can be found in Table S3 (Additional file 1).

**Table S3.** Average age of sampled female Tasmanian devils at each site according to reproductive status.

|  | Lactating | Non-Lactating |
| --- | --- | --- |
| Buckland | 3 | 1.5 |
| Fentonbury | 2.1 | 1.1 |
| Kempton | 2 | 1.1 |
| Narawntapu National Park | 2.7 | 1 |
| Stony Head | 2 | 1 |
| Group Mean | **2.4** | **1.1** |


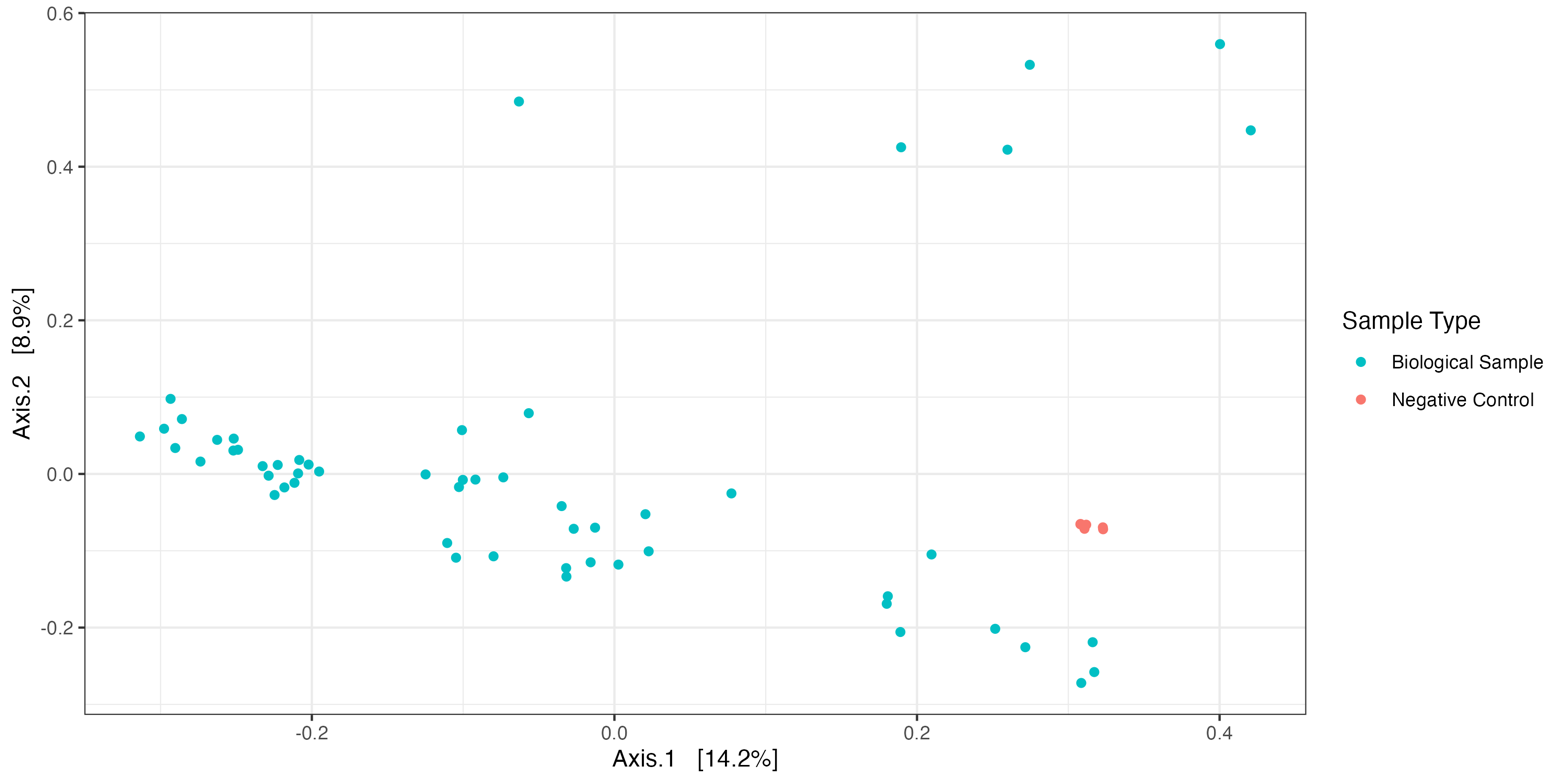


**Figure S3.** PCoA plot of negative controls and biological samples to determine the amount of variation explained by sample type. Negative controls cluster together, indicating community distinctiveness.


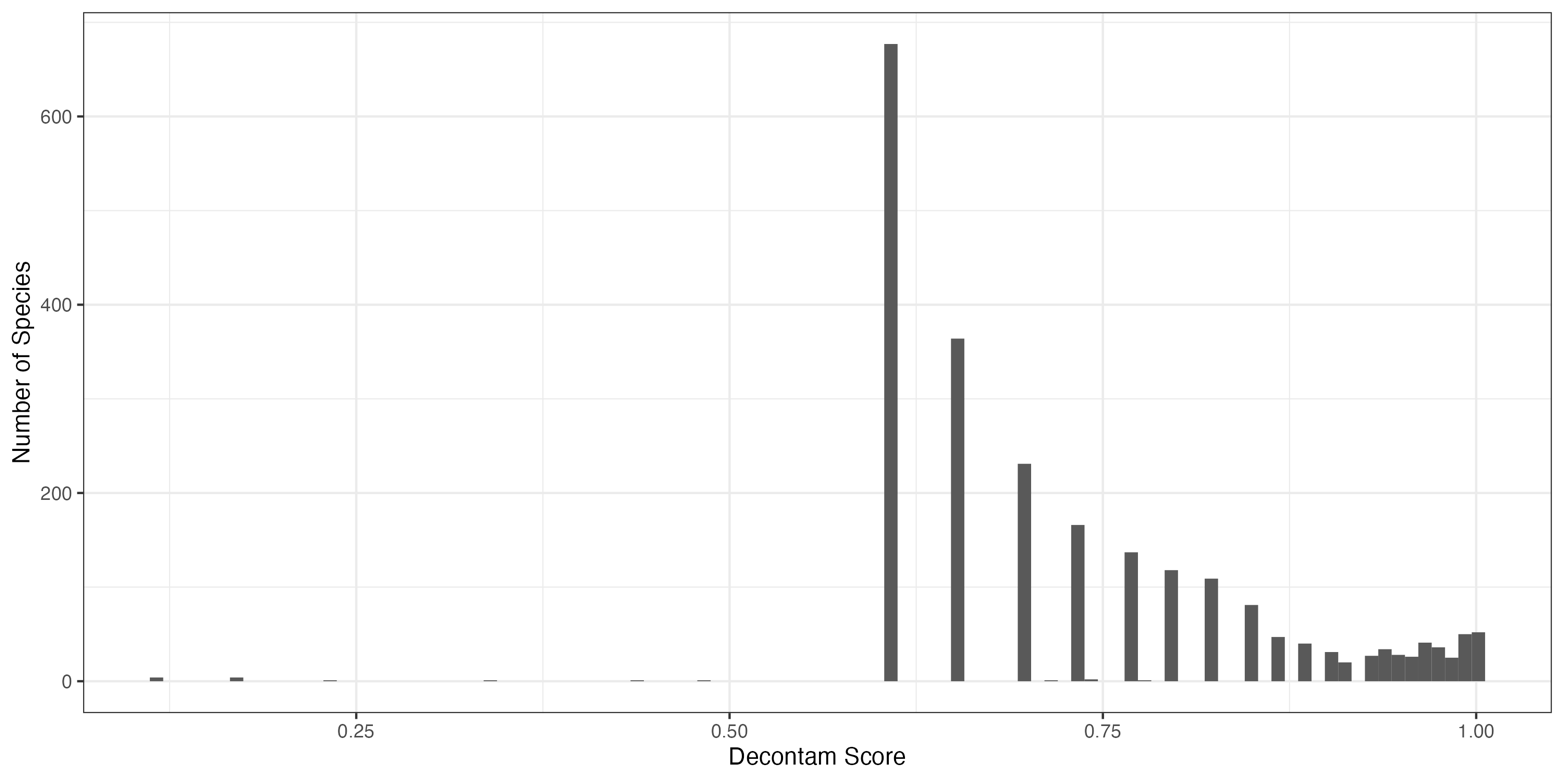


**Figure S4.** Histogram of decontam scores and the prevalence of each score across ASVs. Features below the threshold of 0.5 were subsequently removed prior to additional analysis.


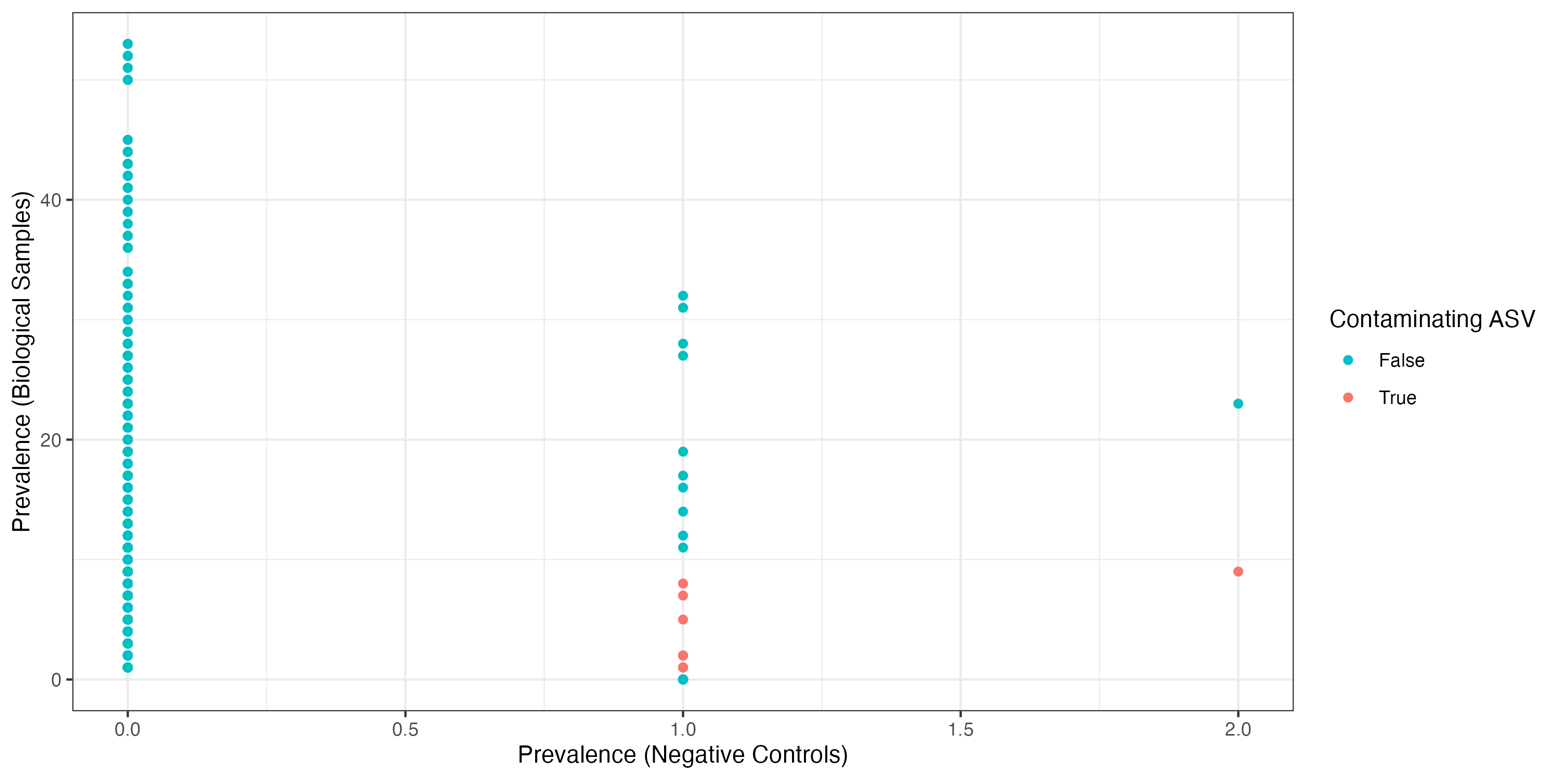


**Figure S5.** Prevalence of contaminating taxa in negative controls and biological samples. Red points indicate features identified in negative controls while blue points indicate features identified in biological samples.

**Table S4.** Summary of amplicon sequence variants (ASVs) identified as contaminants by decontam. These taxa were subsequently removed from the dataset prior to downstream analysis.

| ASV | Kingdom | Phylum | Class | Order | Family | Genus | Species |
| --- | --- | --- | --- | --- | --- | --- | --- |
| ASV3861 | Bacteria | Actinobacteriota | Actinobacteria | Propionibacteriales | Propionibacteriaceae | Cutibacterium | NA |
| ASV3867 | Bacteria | Actinobacteriota | Actinobacteria | Propionibacteriales | Propionibacteriaceae | Pseudopropionibacterium | NA |
| ASV8600 | Bacteria | Proteobacteria | Gammaproteobacteria | Burkholderiales | Burkholderiaceae | Burkholderia Caballeronia Paraburkholderia | NA |
| ASV8602 | Bacteria | Proteobacteria | Gammaproteobacteria | Burkholderiales | Burkholderiaceae | Burkholderia Caballeronia Paraburkholderia | Caballeronia humi |
| ASV8603 | Bacteria | Proteobacteria | Gammaproteobacteria | Burkholderiales | Burkholderiaceae | Burkholderia Caballeronia Paraburkholderia | NA |
| ASV9866 | Bacteria | Proteobacteria | Gammaproteobacteria | Enterobacterales | Yersiniaceae | Serratia | NA |
| ASV9868 | Bacteria | Proteobacteria | Gammaproteobacteria | Enterobacterales | Yersiniaceae | Serratia | NA |
| ASV9870 | Bacteria | Proteobacteria | Gammaproteobacteria | Enterobacterales | Yersiniaceae | Serratia | NA |
| ASV9876 | Bacteria | Proteobacteria | Gammaproteobacteria | Enterobacterales | Enterobacteriaceae | Plesiomonas | NA |
| ASV10380 | Bacteria | Proteobacteria | Gammaproteobacteria | Pseudomonadales | Pseudomonadaceae | Pseudomonas | NA |
| ASV10398 | Bacteria | Proteobacteria | Gammaproteobacteria | Pseudomonadales | Pseudomonadaceae | Pseudomonas | Pseudomonas  psychrophila |
| ASV15538 | Bacteria | Firmicutes | Bacilli | Lactobacillales | Streptococcaceae | Streptococcus | NA |

**Table S5.** Results from generalised linear models testing the correlation between reproductive status and alpha diversity metrics. Location was included as a covariate. Intercept represents reference coefficients 'Lactating' and 'Buckland'. *p* < 0.05 (*), *p* < 0.005 (**), *p* < 0.001 (***).

| Coefficients | Estimate | Std. Error | t value | *p*-value |
| --- | --- | --- | --- | --- |
| 1. *Number of observed ASVs per reproductive status, with location as a covariate* | | | | |
| (Intercept) | 262.75 | 150.93 | 1.741 | 0.08799 |
| Reproductive Status; Non-Lactating | 250.57 | 84.30 | 2.972 | 0.00457 ** |
| Location; Fentonbury | 53.19 | 154.72 | 0.344 | 0.73247 |
| Location; Kempton | 60.34 | 156.75 | 0.385 | 0.70192 |
| Location; Narawntapu | 344.90 | 165.43 | 2.085 | 0.04232 * |
| Location; Stony Head | 124.40 | 190.96 | 0.651 | 0.51780 |
| 1. *Shannon Diversity Index per reproductive status, with location as a covariate* | | | | |
| (Intercept) | 4.345667 | 0.473949 | 9.169 | 3.31e-12 *** |
| Reproductive Status; Non-Lactating | 0.559993 | 0.264720 | 2.115 | 0.0395 * |
| Location; Fentonbury | 0.006059 | 0.485823 | 0.012 | 0.9901 |
| Location; Kempton | 0.258980 | 0.492206 | 0.526 | 0.6011 |
| Location; Narawntapu | 0.312813 | 0.219480 | 0.602 | 0.5498 |
| Location; Stony Head | 0.177144 | 0.599631 | 0.295 | 0.7689 |
| 1. *Faith’s Phylogenetic Diversity per reproductive status, with location as a covariate* | | | | |
| (Intercept) | 10.901 | 7.434 | 1.466 | 0.14896 |
| Reproductive Status; Non-Lactating | 11.634 | 4.152 | 2.802 | 0.00726 ** |
| Location; Fentonbury | 3.407 | 7.621 | 0.447 | 0.65680 |
| Location; Kempton | 3.101 | 7.721 | 0.402 | 0.68969 |
| Location; Narawntapu | 19.222 | 8.148 | 2.359 | 0.02236 * |
| Location; Stony Head | 8.885 | 9.406 | 0.945 | 0.34948 |

**Table S6.** Permutational multivariate variance of analysis (PERMANOVA) results of unweighted and weighted UniFrac distances. *p* < 0.05 (*), *p* < 0.005 (**), *p* < 0.001 (***).

|  | df | Sum of Squares | R^2^ | F Statistic | *p*-value |
| --- | --- | --- | --- | --- | --- |
| 1. *Unweighted UniFrac Distance* | | | | | |
| Reproductive Status | 1 | 0.5021 | 0.02154 | 1.843 | 0.017 ** |
| Location | 4 | 2.0358 | 0.08734 | 1.2006 | 0.001 *** |
| Residual | 49 | 20.7722 | 0.89112 |  |  |
| Total | 54 | 23.3101 | 1.00000 |  |  |
| 1. *Weighted UniFrac Distance* | | | | | |
| Reproductive Status | 1 | 0.0001272 | 0.01531 | 0.8706 | 0.580 |
| Location | 4 | 0.0010245 | 0.12326 | 1.7528 | 0.002 ** |
| Residual | 49 | 0.0071603 | 0.86144 |  |  |
| Total | 54 | 0.0083121 | 1.00000 |  |  |

**Table S7.** Permutation tests to determine if the assumption of homogeneous dispersion effects is met for Permutational Multivariate Analysis of Variance (PERMANOVA) analysis. Comparisons of dispersion effects were made individually between each variable and beta diversity metric. The beta diversity metrics analysed were unweighted and weighted UniFrac distances. p < 0.05 (*), p < 0.005 (**), p < 0.001 (***).

|  | df | Sum of Squares | Mean Square | F Statistic | Number of Permutations | *p*-value |
| --- | --- | --- | --- | --- | --- | --- |
| 1. *Permutation test for homogeneity of multivariate dispersions of unweighted UniFrac distances - reproductive status* | | | | | | |
| Groups | 1 | 0.004127 | 0.0041268 | 5.8583 | 999 | 0.016 * |
| Residuals | 53 | 0.037335 | 0.0007044 |  |  |  |
| 1. *Permutation test for homogeneity of multivariate dispersions of unweighted UniFrac distances - location* | | | | | | |
| Groups | 4 | 0.025239 | 0.0063098 | 8.3098 | 999 | 0.001 *** |
| Residuals | 50 | 0.037966 | 0.0007593 |  |  |  |
| 1. *Permutation test for homogeneity of multivariate dispersions of weighted UniFrac distances - reproductive status* | | | | | | |
| Groups | 1 | 0.00009202 | 0.000092017 | 3.671 | 999 | 0.065 |
| Residuals | 53 | 0.00132849 | 0.000025066 |  |  |  |
| 1. *Permutation test for homogeneity of multivariate dispersions of weighted UniFrac distances - location* | | | | | | |
| Groups | 1 | 0.00011378 | 0.000028444 | 1.1512 | 999 | 0.346 |
| Residuals | 53 | 0.00123542 | 0.000024708 |  |  |  |

**
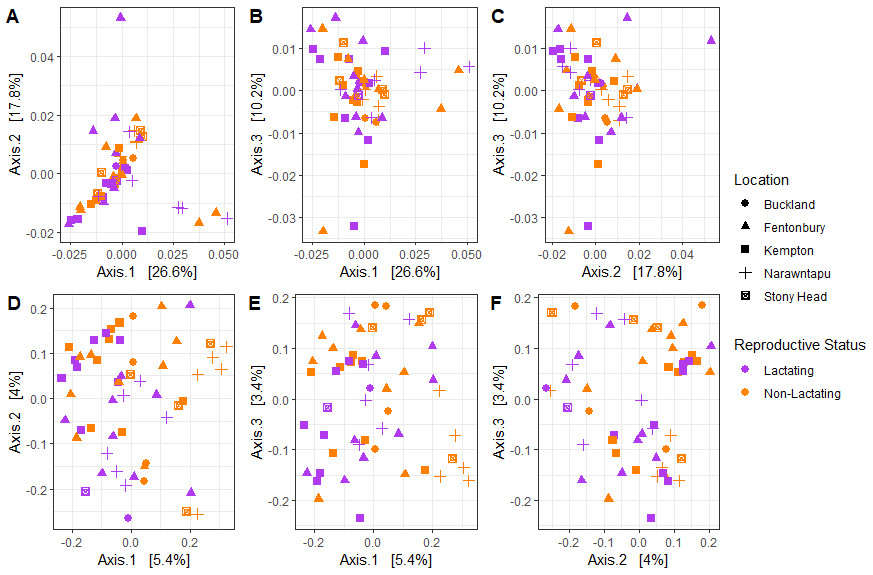
Figure S6.** PCoA ordination plots of (A-C) unweighted and (D-F) weighted UniFrac distances across three axes.

**Table S8.** Permutational multivariate variance of analysis (PERMANOVA) results of unweighted and weighted UniFrac distances between reproductive statuses at each site individually. Only interactions at Narawntapu National Park were significant. *p* < 0.05 (*), *p* < 0.005 (**), *p* < 0.001 (***).

|  | df | Sum of Squares | R^2^ | F Statistic | Number of Permutations | *p*-value |
| --- | --- | --- | --- | --- | --- | --- |
| 1. *Narawntapu National Park - Unweighted UniFrac Distance* | | | | | | |
| Reproductive Status | 1 | 0.5915 | 0.13493 | 1.4038 | 999 | 0.006 ** |
| Residuals | 9 | 3.7921 | 0.86507 |  |  |  |
| 1. *Narawntapu National Park - Weighted UniFrac Distance* | | | | | | |
| Reproductive Status | 1 | 0.0022718 | 0.22058 | 2.547 | 999 | 0.019 * |
| Residuals | 9 | 0.0080276 | 0.77942 |  |  |  |
| 1. *Kempton - Unweighted UniFrac Distance* | | | | | | |
| Reproductive Status | 1 | 0.3979 | 0.07116 | 1.0726 | 999 | 0.118 |
| Residuals | 14 | 5.1939 | 0.92884 |  |  |  |
| 1. *Kempton - Weighted UniFrac Distance* | | | | | | |
| Reproductive Status | 1 | 0.0014192 | 0.07592 | 1.13145 | 999 | 0.249 |
| Residuals | 14 | 0.0080276 | 0.77942 |  |  |  |
| 1. *Fentonbury - Unweighted UniFrac Distance* | | | | | | |
| Reproductive Status | 1 | 0.4449 | 0.06448 | 1.1027 | 999 | 0.113 |
| Residuals | 16 | 6.4560 | 0.93552 |  |  |  |
| 1. *Fentonbury - Weighted UniFrac Distance* | | | | | | |
| Reproductive Status | 1 | 0.0014192 | 0.07592 | 1.3145 | 999 | 0.249 |
| Residuals | 16 | 0.0172748 | 0.92408 |  |  |  |

**
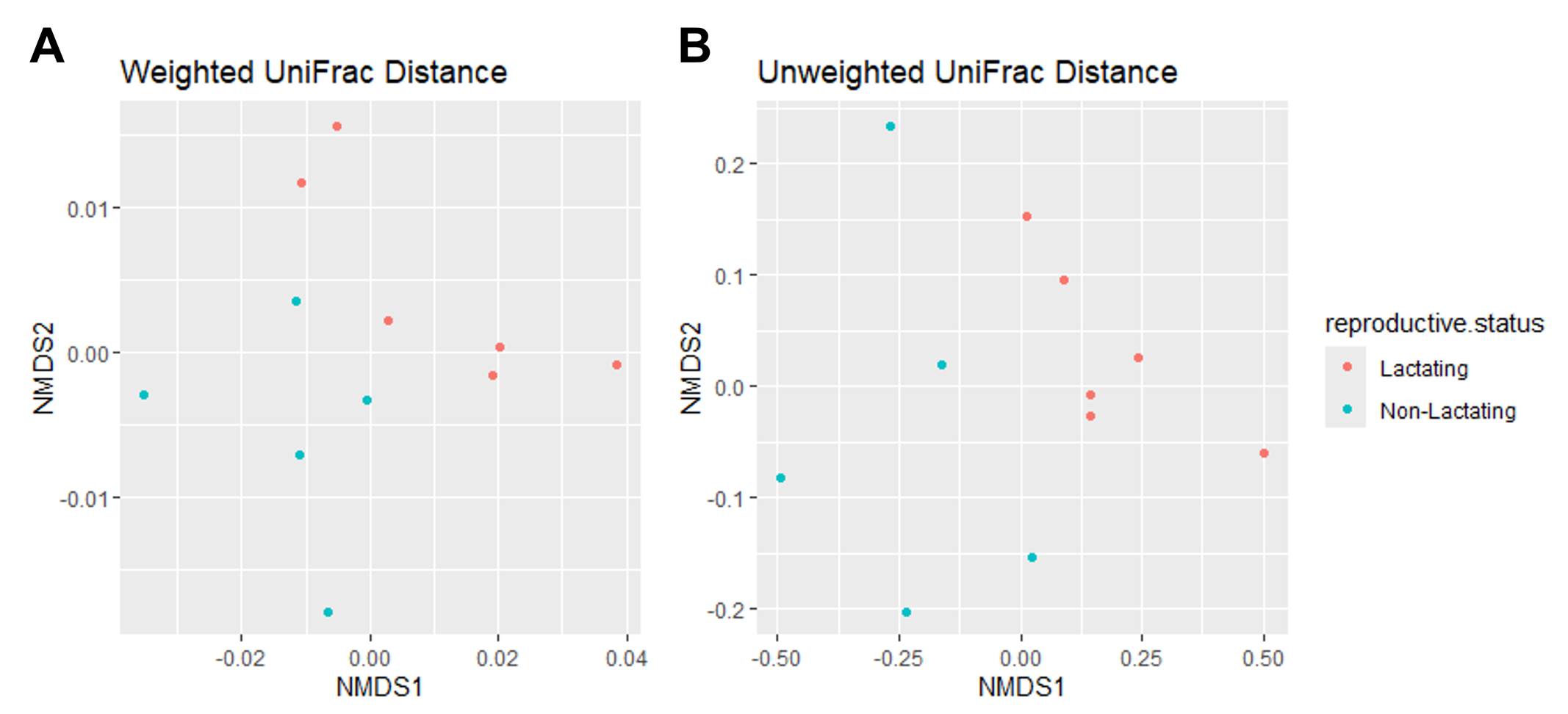
**

**Figure S7.** PCoA ordination plots of weighted (A) and unweighted (B) UniFrac distances by reproductive status at Narawntapu National Park.

**Table S9.** Results from DESeq2 differential abundance analysis between lactating and non-lactating Tasmanian devil pouches. ‘BaseMean’ is the mean of total normalised counts per taxa, ‘log2FoldChange’ indicates the effect size estimate, ‘padj’ are Wald test p-values adjusted for false discovery rate using the Benjamini-Hochberg procedure, while ‘Taxonomic Annotation’ is the most accurate classification for each taxa based on ‘Taxonomic Rank’. Differentially abundant taxa were identified in lactating versus non-lactating pouches, hence a positive log2FoldChange indicates a higher prevalence in lactating pouches, while a negative log2FoldChange indicates a lower prevalence in lactating pouches. A total of 33 differentially abundant taxonomic groups were identified.

| Taxonomic Rank | BaseMean | log2FoldChange | padj | Taxonomic Annotation |
| --- | --- | --- | --- | --- |
| Order | 7680.59452 | 2.01238823 | 0.01534475 | Staphylococcales |
|  | 8.95025761 | -4.2270081 | 0.02300249 | Pedosphaerales |
|  | 282.90043 | 1.63576225 | 0.01081672 | Corynebacteriales |
|  | 41.5753196 | -2.0372208 | 0.0303934 | Xanthomonadales |
| Family | 7366.70552 | 2.1871083 | 0.01769921 | Staphylococcaceae |
|  | 11.9208818 | -4.7061957 | 0.03103328 | Pedosphaeraceae |
|  | 32.0699888 | -2.9259316 | 0.01769921 | Rhodanobacteraceae |
| Genus | 9.14960353 | -22.680489 | 5.81E-14 | *Geobacillus* |
| Amplicon Sequence Variant | 12.4070462 | -23.170653 | 1.39E-16 | Unclassified *Vagococcus* sp. I |
|  | 8.74613501 | -22.680771 | 5.37E-16 | Unclassified *Peptostreptococcus* sp. |
|  | 9.6186282 | -20.402099 | 2.42E-11 | *Wohlfahrtiimonas chitiniclastica* |
|  | 22.1380745 | -21.976663 | 1.26E-18 | Unclassified *Tissierella* sp. I |
|  | 25.0320675 | 25.2654243 | 5.29E-20 | Unclassified *Candidatus Bacilloplasma* sp. I |
|  | 13.2740278 | 24.6901076 | 4.79E-22 | Unclassified *Alloiococcus* sp. I |
|  | 13.3229062 | -23.270656 | 1.11E-18 | *Clostridium colicanis* |
|  | 12.1617669 | -23.139595 | 1.26E-18 | Unclassified *Vagococcus* sp. II |
|  | 11.8012068 | 24.4842313 | 2.11E-19 | *Corynebacterium urealytic*um |
|  | 10.2637142 | 24.3336299 | 2.94E-19 | *Clostridium botulinum* |
|  | 10.3440115 | 24.355744 | 5.33E-17 | Unclassified *Alloiococcus* sp. II |
|  | 7.8611382 | -22.53449 | 6.16E-18 | *Ignatzschineria indica* |
|  | 7.89903106 | -22.541657 | 7.13E-16 | Unclassified *W5053* sp. |
|  | 77.706451 | -10.47301 | 2.32E-07 | Unclassified *Cetobacterium* sp. II |
|  | 72.2946451 | -11.858554 | 1.05E-08 | Unclassified *Cetobacterium* sp. I |
|  | 13.8215647 | 22.7437814 | 5.33E-17 | *Clostridium paraputrificum* |
|  | 15.8275706 | -23.511545 | 5.33E-17 | Unclassified *Tissierella* sp. II |
|  | 17.7196292 | 25.0796068 | 2.30E-29 | Unclassified *Nosocomiicoccus* sp. |
|  | 21.257916 | 25.330276 | 4.42E-18 | Unclassified *Candidatus Bacilloplasma* sp. II |
|  | 7.67890385 | -22.502145 | 8.07E-14 | Unclassified *Moraxella* sp. II |
|  | 5.57250056 | -20.083087 | 1.28E-12 | Unclassified *Tissierella* sp. III |
|  | 5.57001476 | -22.050765 | 3.65E-15 | Unclassified *Tissierella* sp. IV |
|  | 13.0975345 | 24.6592521 | 1.67E-19 | Unclassified *Psychrobacter* sp. I |
|  | 11.2813996 | 24.4483399 | 2.11E-19 | Unclassified *Psychrobacter* sp. II |
|  | 10.9818637 | -21.547679 | 2.04E-16 | Unclassified *Moraxella* sp. I |
