# Supplementary material for: Characterising the Tasmanian devil (Sarcophilus harrisii) pouch microbiome in lactating and non-lactating females
Source: Sci Rep. 2024 Jul 2;14:15188. doi: 10.1038/s41598-024-66097-8 (PMC11220038; doi:10.1038/s41598-024-66097-8)

**Supplementary Methods: In-House Polymerase Chain Reactions**

Prior to continuing extraction protocols and commencing sequencing, in-house polymerase chain reactions (PCR) were performed on a subset of six samples to ensure that amplifiable bacterial DNA was present. The presence of DNA in the resulting PCR products for this subset of samples was confirmed using a 1% agarose gel plate (Additional file 2: Figure S1). PCR was performed using the MyTaq ™ DNA Polymerase kit (Meridian Bioscience, Cincinnati, OH, USA) in a total volume of 50 µL. This contained 3 µL undiluted template DNA at a concentration ranging between 2 – 10 ng/uL, 1 µL each of 20 µM forward and reverse V3-V4 primers, 1 µL MyTaq DNA Polymerase (5 U/µL), 10 µL 5x MyTaq Reaction Buffer (comprised of 5 mM dNTPs and 15 mM MgCl_2_), and 35 µL ddH_2_O. PCR products were amplified using a T100 Thermal Cycler (Bio-Rad, Hercules, CA, USA) with 1 minute at 95˚C for initial denaturation, followed by 30 cycles of 15 seconds denaturation at 95˚C, 15 seconds annealing at 55˚C, and 10 seconds extension at 72˚C.

**Figure S1.** 1% TBE agarose gel stained with SYBR safe, showing 16S rRNA polymerase chain reaction products to confirm presence of bacterial DNA in DNA extraction products. Lane 1 contains a 1 kb ladder, while lanes 2 - 7 consist of bacterial DNA extracted from pouch swabs taken from six female devils.


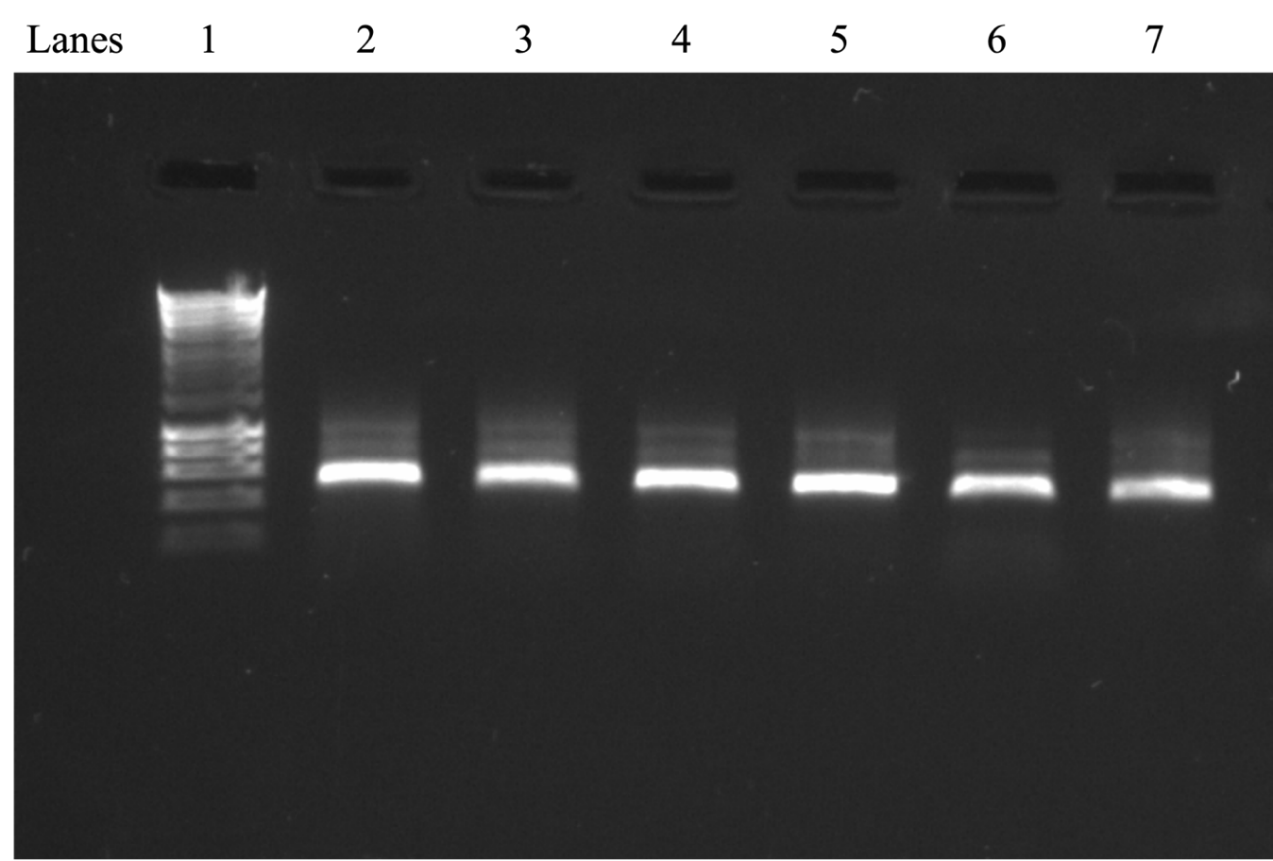

Supplement: Supplementary file 2 — Supplementary Information 2. [file 41598_2024_66097_MOESM2_ESM.docx]
